# Moonlighting Functions of Mammalian Peroxiredoxins in Cellular Signaling

**DOI:** 10.3390/antiox15020231

**Published:** 2026-02-10

**Authors:** Yosup Kim, Eun-Kyung Kim, Ho Hee Jang

**Affiliations:** 1 Department of Health Sciences and Technology, Graduate School of Medicine, Gachon University, Incheon 21999, Republic of Korea; youandkys@gachon.ac.kr (Y.K.); ekkim@gachon.ac.kr (E.-K.K.); 2 Department of Biochemistry, College of Medicine, Gachon University, Incheon 21999, Republic of Korea

**Keywords:** peroxiredoxin, reactive oxygen species, post-translational modifications, antioxidant enzymes, peroxidase activity, chaperone, protein interaction

## Abstract

Peroxiredoxins (Prdxs) are a family of thiol-specific peroxidases that play a central role in maintaining intracellular redox homeostasis. In addition to their classical antioxidant activities, Prdxs function as peroxide sensors, modulators of redox signaling, and molecular chaperones. In this review, we summarize the peroxide-reducing activity, their redox-switch mechanism driven by reversible hyperoxidation, and the chaperone function that arises through oligomerization and accompanying structural changes. We also highlight that the Prdx1–Prdx6 isoforms exhibit distinct subcellular localizations and perform isoform-specific functions, thereby contributing to a wide range of physiological and pathological processes. Furthermore, we compile recent findings showing that diverse post-translational modifications (PTMs), including phosphorylation, acetylation, ubiquitination, glutathionylation, sumoylation, and S-nitrosylation, not only regulate Prdx activity but also contribute to cellular signaling processes. Overall, this review emphasizes that Prdxs are more than simple antioxidant enzymes: they serve as guardians of cellular redox balance and dynamic regulators of signaling networks, underscoring their potential as disease biomarkers and therapeutic targets.

## 1. Introduction

Hydrogen peroxide (H_2_O_2_) and organic hydroperoxides are reactive oxygen species (ROS), whereas peroxynitrite (ONOO^−^) is a reactive nitrogen species (RNS) [1]. These ROS/RNS play roles in physiological processes, including signal transduction, immune responses, and the regulation of cell proliferation and death [2]. However, the excessive accumulation of these oxidants promotes cellular damage and contributes to disease pathogenesis [3].

Peroxiredoxins (Prdxs) are thiol-specific antioxidant proteins that are conserved across eukaryotes, prokaryotes, and plants [4,5,6]. Prdxs serve as the primary line of defense against oxidative stress by sensing and detoxifying these oxidants. In addition to maintaining redox homeostasis, Prdxs function as peroxide sensors, modulators of redox signaling, and molecular chaperones, thereby acting as key regulators that fine-tune complex cellular responses [7,8,9].

Mammals express six Prdx isoforms (Prdx1–Prdx6), which localize to distinct subcellular compartments, including the cytosol, nucleus, mitochondria, endoplasmic reticulum, and peroxisomes, and carry out isoform-specific biological functions [10,11]. Their activities are modulated by diverse post-translational modifications (PTMs), and individual isoforms can be selectively activated or inhibited under pathological conditions, such as cancer, inflammation, aging, and neurodegeneration [12,13,14]. Collectively, these properties highlight the potential use of Prdxs as disease biomarkers and therapeutic targets [15,16,17,18].

This review provides an overview of Prdx biology, covering their antioxidant activity, roles as a peroxide sensor and modulator of redox signaling, oxidation–reduction switching mechanisms, chaperone functions driven by oligomerization and associated structural transitions, and compartment- and isoform-specific functions in cells. Finally, we summarize recent studies on diverse PTMs of Prdxs and discuss how these modifications regulate cellular functions and influence biological outcomes, thereby highlighting the biological significance and biomedical potential of the Prdx family.

## 2. Major Functions and Biological Roles of Prdxs

Prdxs are widely recognized for their primary role in protecting cells from oxidative damage. However, accumulating evidence indicates that, beyond their canonical antioxidant function, certain Prdxs act as peroxide sensors that detect changes in the intracellular redox state or function as molecular chaperones, thereby regulating diverse cellular processes [10]. These multifaceted functions of Prdxs contribute to the modulation of distinct pathological programs, ranging from metabolic reprogramming and immune evasion within the tumor microenvironment to the maintenance of structural and redox homeostasis in muscle and nervous systems [17,19,20,21]. In this section, we summarize the characteristics and functions of Prdx1 through Prdx6, with particular emphasis on their disease-associated roles.

### 2.1. Peroxidase Activity

Prdxs constitute a family of thiol-dependent peroxidases that are classified into three subgroups based on the number of cysteine residues involved in catalysis and the disulfide bond formed: typical 2-Cys (Prdx1–4), atypical 2-Cys (Prdx5), and 1-Cys (Prdx6), comprising a total of six mammalian isoforms [22]. Prdx1 and Prdx2 are primarily localized to the cytosol, nucleus, and plasma membrane. Prdx3 is enriched in mitochondria and serves as a key regulator of mitochondrial ROS homeostasis [23]. Recent studies have suggested that Prdx3 is dually localized within mitochondrial subcompartments, including both the intermembrane space and matrix [24]. Prdx4 is mainly localized in the endoplasmic reticulum (ER) and is also present in the extracellular space. In addition, a testis-specific Prdx4 variant, Prdx4t, has been identified, which lacks exon 1 of canonical Prdx4 and is proposed to protect testicular cells from oxidative stress [25]. Prdx5 shows a multicompartment distribution, including the cytosol, mitochondria, and peroxisomes. Prdx6, the only mammalian 1-Cys Prdx, is predominantly localized in the cytosol, lysosome and is associated with cellular membranes (Table 1) [9,26].

**Figure 1:**
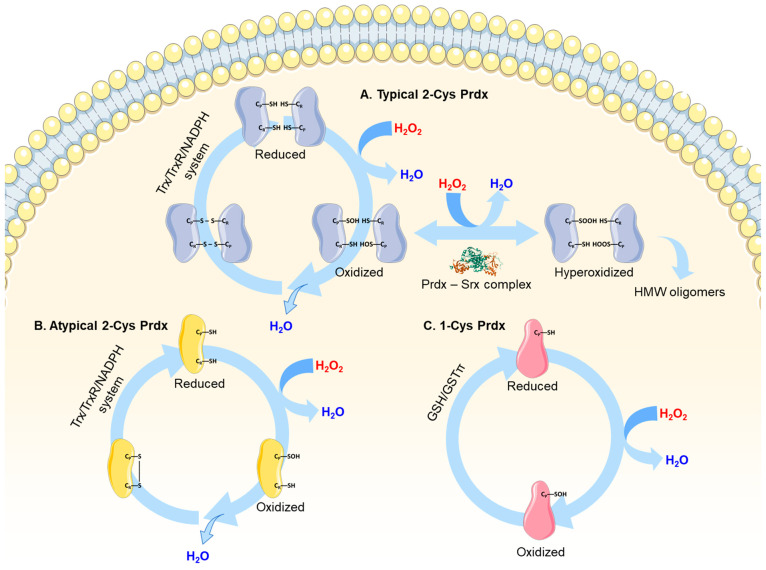
The catalytic cycle of typical 2-Cys, atypical 2-Cys, and 1-Cys Prdxs. (A) Reduced typical 2-Cys Prdxs exist with thiol active sites (–SH). Upon exposure to H_2_O_2_, the C_P_ is oxidized to –SOH, which is then resolved by the resolving cysteine (C_R_) to form an intersubunit disulfide (S–S); this peroxidase cycle yields H_2_O. The Trx/TrxR/NADPH system reduces the disulfide and regenerates the active enzyme. Under excess peroxide, C_P_ becomes hyperoxidized to –SO_2_H, inhibiting peroxidase activity and promoting assembly into high-molecular-weight (HMW) oligomers with holdase chaperone function. Sulfiredoxin (Srx) forms a transient Prdx–Srx complex to specifically reduce the sulfinic form and return Prdx to the catalytic cycle [35]. (B) Reduced atypical 2-Cys Prdxs contain both the C_P_ and the C_R_ within the same polypeptide chain. Upon reaction with H_2_O_2_, C_P_ is oxidized to –SOH, which subsequently forms an intramolecular disulfide bond with C_R_. This reaction converts H_2_O_2_ to H_2_O and completes the peroxidase cycle. The oxidized enzyme is then reduced by the Trx/TrxR/NADPH system, restoring the reduced and catalytically active form. (C) 1-Cys Prdxs possess only the C_P_ and lack a C_R_. Following oxidation of C_P_ to –SOH by H_2_O_2_, the oxidized cysteine cannot form a disulfide bond within the enzyme. Instead, regeneration of the reduced active form is mediated by GSH-dependent systems, including GSH and GSTπ. Through this mechanism, 1-Cys Prdxs complete the catalytic cycle while maintaining peroxide-reducing activity. (PDB codes: Prdx-Srx complex, 2RII [8]).

Despite their localizations and structural differences, all Prdxs share a conserved peroxidatic cysteine (C_P_) at the active site, which reacts with high efficiency with ROS and RNS, thereby functioning as major intracellular peroxide scavengers. Upon exposure to H_2_O_2_, the C_P_ of typical 2-Cys Prdxs (Prdx1–4) is oxidized to cysteine-sulfenic acid (C_P_–SOH), which is then resolved by the resolving cysteine (C_R_) to form an intermolecular disulfide (S–S) within the homodimer; this peroxidase cycle yields H_2_O. The thioredoxin–thioredoxin reductase (Trx–TrxR) system reduces the disulfide and regenerates the active enzyme. [12,27,28]. Notably, typical 2-Cys Prdxs harbor conserved sequence motifs, including the Gly-Gly-Leu-Gly (GGLG) motif and the C-terminal Tyr-Phe (YF) motif, which are critical for their unique redox properties. The GGLG motif confers structural flexibility around the C_P_, ensuring its exposure for efficient peroxide access and catalysis. In contrast, the YF motif stabilizes the C-terminal region in a manner that creates steric hindrance, thereby hindering disulfide bond formation between C_P_ and C_R_ of the adjacent subunit. This delayed resolution promotes conformational rearrangements required for hyperoxidation-dependent oligomerization. Together, these motifs underlie the pronounced sensitivity of typical 2-Cys Prdxs to hyperoxidation and enable their functional transition from peroxidases to redox signaling mediators and molecular chaperones under oxidative stress [29,30,31]. In contrast, atypical 2-Cys Prdx5 forms an intramolecular disulfide bond between its C_P_ and C_R_ within a single polypeptide chain, reflecting a distinct redox-cycling mechanism. Prdx5 is also reduced back to its catalytically active state by the Trx–TrxR system, allowing continuous catalytic turnover [17]. Unlike the typical 2-Cys Prdxs, Prdx5 lacks the GGLG and YF motifs, which makes it much harder to be inactivated by excess peroxide. This allows it to keep working steadily as an antioxidant in various parts of the cell, such as the mitochondria and peroxisomes [32]. Similar to other isoforms, 1-Cys Prdx6 reacts with H_2_O_2_ at its active site, leading to the oxidation of C_P_ to C_P_–SOH. However, Prdx6 lacks C_R_, GGLG, and YF motifs and therefore cannot form disulfide intermediates within the enzyme. Instead, the C_P_ of Prdx6 is regenerated through a glutathione (GSH)-dependent system, in which GSH S-transferase π (GSTπ) acts as a key mediator. This mechanism enables Prdx6 to function as a GSH peroxidase (GPx) by reducing phospholipid hydroperoxides. Beyond its peroxidase activity, Prdx6 is distinguished by its multifunctional “moonlighting” capabilities, characterized by a unique catalytic triad (Ser32, His26, Asp140) that confers acidic Ca^2+^-independent phospholipase A_2_ (aiPLA_2_) activity [33]. This enzymatic function, complemented by lysophosphatidylcholine acyltransferase activity, enables Prdx6 to play a critical role in pulmonary surfactant metabolism and the removal and repair of oxidatively damaged membrane phospholipids (Figure 1) [34].

### 2.2. Chaperone Activity

The Cys–SOH can be further oxidized to sulfinic acid (Cys–SO_2_H) and, under more severe conditions, to sulfonic acid (Cys–SO_3_H). The sulfinic acid state can be reversed by Srx1 via an adenosine triphosphate (ATP)-dependent mechanism, whereas oxidation to the sulfonic acid state is considered irreversible. Although sulfonylated Prdxs lose their peroxidase activity, they can undergo structural transitions that favor the formation of HMW oligomers and acquisition of chaperone (holdase) activity. This helps protect folding-sensitive client proteins during heat or oxidative stress, supporting proteostasis and promoting cell survival. Thus, structural switching and functional duality are essential components of cellular stress responses [26].

### 2.3. Circadian Rhythm

The oxidation state of Prdxs oscillates in a circadian manner across diverse organisms [5]. Red blood cells express Prdx1, Prdx2, and Prdx6, among which Prdx2 undergoes daily hyperoxidation and exhibits circadian-like oscillations [36]. This phenomenon has been attributed to H_2_O_2_ generated during hemoglobin-mediated oxygen transport, which drives Prdx2 hyperoxidation; the hyperoxidized Prdx2 is then degraded by the 20S proteasome in a periodic manner. Accordingly, the diurnal oscillation of Prdx2-SO_2_H is thought to be regulated not by Srx-dependent reduction but by H_2_O_2_-induced hyperoxidation coupled with proteasomal turnover. Approximately 1% of cellular Prdx2 is estimated to undergo this hyperoxidation–degradation cycle each day, and the overall abundance of Prdx2 gradually decreases as red blood cells age [37].

### 2.4. Damage-Associated Molecular Pattern (DAMP)

Extracellular Prdx1 acts as a DAMP when cells are injured. In mouse models of acute liver injury induced by acetaminophen or carbon tetrachloride, serum Prdx1 increases and promotes inflammation through nuclear factor kappa-light-chain-enhancer of activated b cells and the NOD-, LRR- and pyrin domain-containing protein 3 (NLRP3) inflammasome, enhancing macrophage infiltration and the release of interleukin-1 beta (IL-1β), IL-6, and tumor necrosis factor alpha (TNF-α). Prdx1-deficient mice are protected from such responses, whereas the administration of recombinant Prdx1 restores inflammation [38].

Treatment of colorectal cancer cell lines such as RKO and SW480 with recombinant Prdx1 activates the NLRP3 inflammasome, leading to caspase-1 activation. Activated caspase-1 cleaves gasdermin D (GSDMD) to generate a pore-forming GSDMD-N terminal fragment that permeabilizes the plasma membrane. This process promotes the release of pro-inflammatory cytokines such as IL-1β and IL-18 and drives tumor cell destruction, thereby suppressing tumor growth and metastasis. Thus, in addition to its intracellular antioxidant and signaling roles, Prdx1 functions as an extracellular inflammatory mediator [39].

### 2.5. Mitophagy Regulator

Prdx3 is a key regulator of mitophagy in cardiomyocytes. Under hypoxia–reoxygenation stress, Prdx3 deficiency leads to mitochondrial H_2_O_2_ accumulation, which promotes excessive mitochondrial fission and disrupts mitophagy, ultimately resulting in cell death. Although hypoxia–reoxygenation alone or Prdx3 deficiency alone increases the expression of mitophagy regulators such as Parkin and BNIP3, their expression is paradoxically suppressed when hypoxia–reoxygenation occurs in Prdx3-deficient cells. This pattern suggests the impaired clearance and progressive accumulation of damaged mitochondria. In conjunction with defective lysosomal acidification, these defects accelerate cardiomyocyte death. Collectively, these findings indicate that Prdx3 preserves mitochondrial quality control by limiting oxidative mitochondrial injury and coordinating mitochondrial dynamics, mitophagy, and lysosomal integrity, thereby protecting the heart from hypoxia–reoxygenation-induced injury [40].

### 2.6. Lactylation-Associated Marker

Integrated multi-omics analyses have identified Prdx1 as a lactylation-related gene in breast cancer. Prdx1 was selectively overexpressed in tumor-infiltrating monocytes, and increased interactions between Prdx1-positive monocytes and fibroblasts were observed in the breast tumor microenvironment. These findings support a model in which Prdx1 contributes to breast cancer development and progression by shaping lactylation-associated regulatory programs and promoting immune evasion [41].

### 2.7. Redox Signal Amplifier

During exercise, muscle contractions generate H_2_O_2_, which is essential for muscle adaptation and mitochondrial biogenesis. Prdx2 amplifies this ROS cue and couples it to transcriptional outputs, effectively functioning as a signal “amplifier.” In Prdx2-deficient myotubes, exercise-mimetic stimulation fails to induce the upregulation of mitochondrial oxidative phosphorylation-associated genes, including cyclooxygenase 1 (COX1), COX2, COX3, and ATP6. These observations indicate that the redox-dependent transcriptional programs underlying exercise-induced increases in mitochondrial capacity in human skeletal muscle myotubes require Prdx2 to transduce the effects of H_2_O_2_ [20]. A related concept, redox stress response resistance, has been proposed in the context of aging, with Prdx2 positioned as a central determinant. Analogous to insulin resistance, aged cells exhibit an impaired ability to activate antioxidant and cytoprotective responses despite elevated ROS levels. This phenotype has been attributed to chronic oxidative stress-driven hyperoxidation and Prdx2 inactivation. Consequently, Prdx2-mediated redox relay signaling is disrupted, preventing the engagement of adaptive defense mechanisms and predisposing cells to death under oxidative stress [42].

### 2.8. Reproductive Biomarker

In a clinical cohort of women with tubal or male factor infertility (*n* = 138), follicular fluid samples were collected on the day of oocyte retrieval for in vitro fertilization (IVF) or intracytoplasmic sperm injection, and Prdx4 concentrations were quantified to assess their associations with IVF outcomes. Higher follicular fluid Prdx4 levels are associated with increased clinical pregnancy rates. Moreover, the highest Prdx4 group showed significantly higher fertilization, clinical pregnancy, and live birth rates than the lowest group [43].

### 2.9. Mitochondrial Protector

In hepatocytes, Prdx1 detoxifies H_2_O_2_, thereby dampening signal transducer and activator of transcription 1/3 (STAT1/3) signaling and preserving mitochondrial function. Palmitic acid directly binds to Prdx1 and suppresses its peroxidase activity, exacerbating non-alcoholic steatohepatitis (NASH). Rosmarinic acid has been proposed as a potent Prdx1 activator that stabilizes peroxidatic cysteine and enhances enzymatic activity, highlighting a potential therapeutic strategy for NASH [44].

Prdx5 promotes the expression of the mitochondrial transport regulators Miro1 and Milton. Accordingly, Prdx5 deficiency impairs mitochondrial trafficking and muscle regeneration, leading to central nuclear aggregation within myofibers and compromised muscle function. Moreover, Prdx5/Prdx3 double-knockout mice exhibit severe sarcopenia and premature muscle aging, suggesting that the complementary and synergistic actions of these two isoforms are essential for maintaining muscle homeostasis and functional integrity [45].

## 3. PTMs of Prdxs

**Figure 2:**
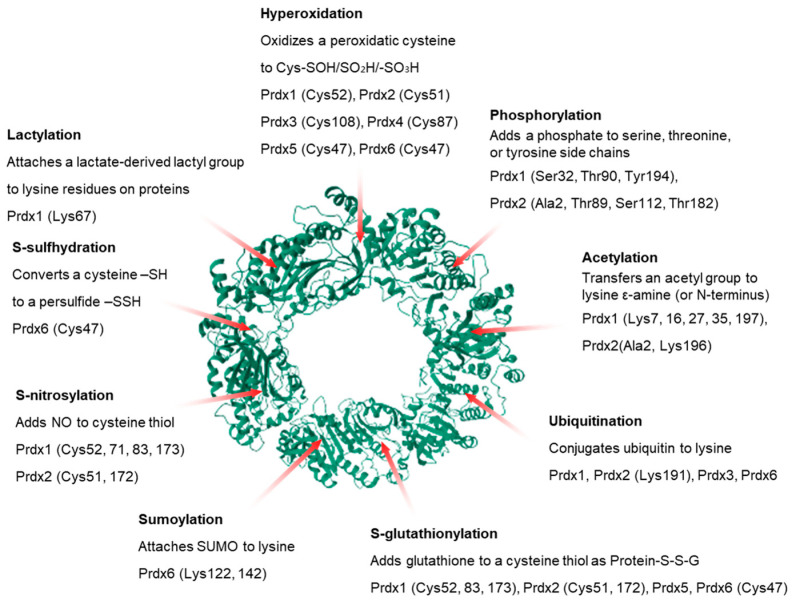
Key post-translational modifications of Prdx isoforms. Site-specific PTMs of Prdxs regulate peroxidase activity, oligomer stability, subcellular localization, degradation pathways, and protein–protein interactions (PDB codes: Human Prdx2, 7KIZ).

Prdxs respond sensitively to changes in the cellular redox environment and gain functional diversity and regulatory flexibility through various PTMs [46]. These modifications determine antioxidant activity, structural stability, protein–protein interactions, and roles within signaling pathways, and they carry particular significance in stress, cancer, and inflammatory disease settings [12,47]. This section summarizes the major PTMs reported to date and discusses their physiological and pathological implications (Figure 2).

### 3.1. Hyperoxidation

Prdxs are highly conserved H_2_O_2_-scavenging peroxidases that maintain strict control over intracellular peroxide levels under physiological conditions. In contrast, under pathological conditions or severe oxidative stress, the catalytic cycle of Prdxs may become overwhelmed, resulting in excessive peroxide-driven oxidation and a reversible or irreversible loss of enzymatic activity [48,49]. Consequently, the biological implications of Prdx inactivation in peroxide-dependent signaling have remained controversial.

An early hypothesis proposed to explain this phenomenon is the floodgate model, which posits that transient inactivation of Prdxs permits localized accumulation of H_2_O_2_, thereby enabling redox-dependent signaling of downstream target proteins [50]. However, subsequent studies have suggested that this model alone is insufficient to fully account for the diverse roles of Prdxs in redox signal regulation. Indeed, accumulating evidence demonstrates that Prdx hyperoxidation represents not merely a loss-of-function event but can actively reprogram Prdx behavior.

Upon oxidation by H_2_O_2_, Prdxs can form Prdx–S–S–Target mixed disulfide intermediates, which mediate the oxidation of target proteins through a redox relay mechanism. This process establishes Prdxs as bona fide redox sensors that catalyze disulfide bond formation in target proteins. Through this mechanism, Prdxs regulate multiple signaling pathways, including those involving Phosphatase and tensin homolog deleted on chromosome 10 [51], STAT3 [52], apoptosis signal-regulating kinase 1 [53], glycerophosphodiester phosphodiesterase 2 [54], apurinic/apyrimidinic endonuclease 1 [55], High Mobility Group Box 1 [56], and Parkinson disease protein 7 [57]. These findings support the notion that some Prdxs function as catalytic redox sensors, mediating redox relay mechanisms that transmit oxidative signals to target proteins via disulfide bond formation (the signal peroxidase model) [53].

Furthermore, Prdx activity can also be regulated by PTMs that are independent of peroxide-mediated oxidation. For example, Src kinase-mediated phosphorylation of Prdx1 at Tyr194 induces transient inactivation of Prdx1 and is associated with localized H_2_O_2_ accumulation in the vicinity of receptor complexes. This localized increase in H_2_O_2_ promotes reversible inactivation of protein tyrosine phosphatases (PTPs) and other oxidation-sensitive signaling proteins, thereby amplifying phosphorylation-dependent signaling cascades [58]. Taken together, these observations suggest that Prdx hyperoxidation operates within a broader regulatory network that extends beyond the classical floodgate model. Rather than functioning solely as redox buffers that are passively inactivated to permit peroxide signaling, Prdxs act as redox sensors whose hyperoxidation modulates enzymatic activity, protein–protein interactions, and cellular stress responses in an isoform- and tissue-specific manner [59].

### 3.2. Phosphorylation

Prdx1 is specifically phosphorylated at Thr90 by the Cdc2–Cyclin B complex, suppressing peroxidase activity by approximately 80%. This allows H_2_O_2_ accumulation during mitosis, inhibits phosphatases such as Cdc25C, maintains Cdc2 activity, and promotes cell division [60]. Upon stimulation by growth factors such as platelet-derived growth factor or epidermal growth factor, lipid-raft–localized receptors activate membrane nicotinamide adenine dinucleotide phosphate oxidase to generate H_2_O_2_. The resulting H_2_O_2_ activates Src-family protein tyrosine kinases (PTKs), leading to the phosphorylation of a small pool (0.2–0.4%) of Prdx1 (Tyr194) and further inhibition of its peroxidase activity. Therefore, local H_2_O_2_ near the membrane was not removed, reinforcing Src activity. In parallel, H_2_O_2_ oxidizes the catalytic cysteines in PTPs, limiting Prdx1 dephosphorylation and sustaining PTK-driven receptor signaling [58].

The CDK5/p35 complex has been identified as a binding partner of Prdx2 and phosphorylates Prdx2 at Thr89 in response to neurotoxic stimuli such as 1-methyl-4-phenylpyridinium. This phosphorylation suppresses the peroxidase activity of Prdx2, thereby diminishing its ROS-scavenging capacity and ultimately promoting the death of dopaminergic neurons. These findings suggest that CDK5-mediated phosphorylation of Prdx2 contributes to the pathophysiology of Parkinson’s disease [61].

### 3.3. Acetylation

Acetylated Prdxs are more stable and retain higher antioxidant activities under oxidizing conditions. However, the deacetylase histone deacetylase 6 (HDAC6) removes acetyl groups from Prdx1 at lysine (Lys) 197 and Prdx2 (Lys196), rendering them more prone to hyperoxidation by H_2_O_2_. The inhibition of HDAC6 (e.g., tubacin or siRNA) increases Prdx acetylation, thereby enhancing H_2_O_2_-reducing activity, improving oxidative tolerance, limiting HMW conversion, and reducing cell death [62]. Similarly, in Alzheimer’s disease models, Amyloid β has been reported to reduce acetylation of Prdx1 at Lys197, leading to impaired antioxidant capacity, increased ROS and intracellular Ca^2+^ levels, and defective axonal transport of mitochondria. These abnormalities were partially rescued by treatment with Tubastatin A selective HDAC6 inhibitor, suggesting that HDAC6 inhibition restores Prdx1 acetylation, thereby improving ROS/Ca^2+^ homeostasis and mitochondrial function [63]. The HDAC6–Prdx1 axis also appears to play a central role in focal cortical dysplasia (FCD), which is a major cause of drug-resistant epilepsy. In the cortical tissue of patients with FCD type II and in a BCNU-induced rat model, HDAC6 expression was elevated, which was associated with reduced Prdx1 acetylation. This decrease in Prdx1 acetylation coincides with increased ROS levels and aggravates oxidative stress. Tubastatin A treatment restored Prdx1 acetylation via HDAC6 inhibition, reduced oxidative stress, decreased seizure frequency, and prolonged seizure latency [64].

In contrast to HDAC6, the Lys acetyltransferase males absent on the first (MOF) acetylates Prdx1 at Lys197, preventing Prdx1 hyperoxidation and preserving its enzymatic activity. Lipopolysaccharide (LPS) decreases MOF abundance and Prdx1 acetylation, thereby increasing intracellular H_2_O_2_ levels. Elevated H_2_O_2_ concurrently activates extracellular signal-regulated kinase 1/2 (ERK1/2) signaling and enhances glycolysis, which in turn promotes the production of the pro-inflammatory cytokine IL-6. Collectively, these findings indicated that the MOF–Prdx1–ERK axis contributes to the activation of inflammatory macrophages [65]. Collectively, these findings support the idea that modulation of Prdx acetylation may represent a promising therapeutic strategy for neurodegenerative disorders and inflammatory macrophage activation.

### 3.4. Ubiquitination

Ubiquitination involves the covalent attachment of ubiquitin to Lys residues and directs proteins toward proteasomal degradation [66]. Under oxidative stress, Prdx1, Prdx2, Prdx3, and Prdx6 undergo oxidation-linked turnover. In Prdx2, oxidation triggers a C-terminal α-helix to loop transition that exposes Lys191 to solvent; Lys191 then becomes a ubiquitination site, targeting Prdx2 for proteasome- or autophagy-mediated degradation [4].

Prdx1 acts as a key antioxidant enzyme in response to nitrosative stress induced by ischemic insult Under pathological conditions; however, it also exerts a dual role by undergoing selective degradation that exacerbates neurovascular injury. Under oxygen–glucose deprivation conditions that mimic ischemia, Although Prdx1 expression is initially upregulated in response to ONOO^−^, it is subsequently ubiquitinated and degraded by the E3 ubiquitin ligase E6AP. This ubiquitination and downregulation of Prdx1 are also observed in the middle cerebral artery occlusion model and are associated with increased nitrotyrosine accumulation and enhanced E6AP activity in vascular endothelial cells [67].

### 3.5. S-Glutathionylation

Glutathionylation is a reversible PTM in which GSH forms a disulfide bond with a protein thiol group [68]. Redox proteomic analyses showed that Prdx1 undergoes glutathionylation under oxidative stress in human T lymphocytes, A549 cells, and HeLa cells. Prdx1 contains four cysteine residues (Cys52, Cys71, Cys83, and Cys173) and glutathionylation has been detected at Cys52, Cys83, and Cys173. Notably, Cys83 is located at the oligomer interface and is rapidly glutathionylated upon H_2_O_2_ exposure, which destabilizes the decameric assembly and promotes its conversion to the dimeric form, thereby weakening chaperone activity [69]. This modification is reversibly regulated by Srx and glutaredoxin1 (Grx1) [70].

Prdx2 contains Cys51, Cys70, and Cys172, and oxidative stress induces glutathionylation at Cys51 and Cys172, leading to the suppression of peroxidase activity. In this context, the GSH/Grx1 system can restore Prdx2 activity and function as an alternative to the Trx–TrxR system [71]. In macrophages exposed to LPS-induced oxidative stress, oxidized Prdx2 released to the extracellular space is secreted in a glutathionylated form, and a similar extracellular release has been observed in human embryonic kidney cells following TNF-α treatment. Extracellular Prdx2 in this state has been implicated in the induction of inflammatory signaling, including TNF-α production [72].

Prdx5 is also reported to undergo glutathionylation in rat hepatocytes during oxidative stress [73].

As the only mammalian 1-Cys Prdx, Prdx6 is inactivated when its peroxidatic cysteine (Cys47) is oxidized, resulting in a loss of peroxidase activity. Whereas typical Prdxs form an intramolecular or intermolecular disulfide via a resolving cysteine and are subsequently reduced by the Trx-TrxR system, Prdx6 lacks a resolving cysteine and therefore cannot use this pathway. Instead, GSTπ acts as a mediator to promote formation of a Prdx6–GSH heterodimer, during which Cys47 becomes glutathionylated. Subsequent reduction with GSH restores Cys47 and reactivates the peroxidase activity of Prdx6 [74].

### 3.6. Sumoylation

Oxidative stress and aging promote aberrant sumoylation, which has been implicated in multiple diseases. Under oxidative stress, Prdx6 is abnormally modified by SUMO1 at Lys122 and Lys142 residues. Sumoylated Prdx6 displays reduced GPx and aiPLA_2_ activities, loses cytoprotective function, and ultimately induces cell death, indicating that Prdx6 sumoylation can be pathogenic under oxidative conditions [75].

### 3.7. S-Nitrosylation

S-nitrosylation is a reversible PTM in which a nitric oxide (NO) group is covalently attached to a cysteine thiol, serving as an important regulatory mechanism in diverse signaling pathways [76]. In Prdx1, S-nitrosylation has been reported at Cys52, Cys83, and Cys173. NO donors such as S-nitrosocysteine (SNOC) and S-nitrosoglutathione (GSNO) nitrosylate Prdx1, promoting formation of a Cys52–Cys173 disulfide, disrupting oligomeric assembly, and suppressing peroxidase activity. This modification is reversed by denitrosylation via the Trx–TrxR system, which restores enzymatic function. This regulation is thought to transiently divert Prdx1 from the peroxidase cycle, enabling broader participation in oxidative stress responses [12,77,78].

In Prdx2, NO modifies the peroxidatic cysteine, suppressing antioxidant activity, and disrupting dimer formation. Consequently, the ROS-scavenging capacity is reduced, weakening the cell’s redox defense. The GSNO induces nitrosylation of Prdx2 at Cys51 and Cys172 (but not Cys70), promoting H_2_O_2_ accumulation. The resulting oxidative burden activates the adenosine monophosphate-activated protein kinase–sirtuin 1 pathway and enhances apoptosis in A549 and NCI-H1299 lung cancer cells [79]. In SH-SY5Y cells treated with SNOC, increased levels of nitrosylated Prdx2 were observed, accompanied by reduced antioxidant function. Similarly, elevated SNO-Prdx2 levels have been detected in rotenone-based Parkinson’s disease models and in brain tissues of patients with Parkinson’s disease, supporting the association between Prdx2 S-nitrosylation and neurodegenerative pathology [80].

### 3.8. S-Sulfhydration (Persulfidation)

Hydrogen sulfide is a gaseous signaling mediator that participates in intracellular signal transduction and can induce a PTM termed S-sulfhydration (also referred to as persulfidation), in which a C_P_ thiol group (–SH) is converted to persulfide (–SSH) [81]. In Prdx2, approximately 15% of C_P_ is basally persulfidated. Upon H_2_O_2_ exposure, the Cys–SSH levels decrease, concomitant with an increase in oxidized persulfide species, including the perthiosulfenic acid derivative (C_P_–SSOH) and the perthiosulfinic form (C_P_–SSO_2_H). TrxR1 knockdown increased the abundance of both C_P_–SSH and C_P_–SSO_2_H, supporting the notion that the Trx–TrxR system participates in the reduction of persulfidated Prdx2 and thereby contributes to C_P_ thiol group protection [82].

In Prdx6, Cys47 can be persulfidated by H_2_S-derived species, which is critical for preserving Prdx6 peroxidase activity and protecting cells from oxidative stress. However, shear stress generated by blood flow activates the transcription factor KLF2, which increases miR-27b expression and consequently suppresses cystathionine γ-lyase (CSE) expression. Reduced CSE expression decreases the production of H_2_S-related species, leading to loss of S-sulfhydration at Prdx6. This shift promotes Prdx6 hyperoxidation and decamerization, and suppresses peroxidase activity, ultimately causing ROS accumulation and membrane lipid peroxidation in endothelial cells and disrupting redox homeostasis [83].

### 3.9. Lactylation

Regorafenib (RGF) is a key second-line therapy for patients with advanced hepatocellular carcinoma (HCC) who experience disease progression after sorafenib treatment; however, acquired resistance frequently develops. CRISPR/Cas9 screening of RGF-treated HCC cells identified zinc finger protein 207 (ZNF207) as the major determinant of regorafenib resistance. Mechanistically, ZNF207 promotes Prdx1 lactylation at Lys67, which enhances nuclear factor erythroid 2–related factor 2 (NRF2) nuclear translocation and transcriptional activation. This ZNF207–Prdx1–NRF2 axis induces an antioxidant program that suppresses ferroptosis under regorafenib pressure, thereby enabling HCC cells to evade cell death and accelerating the development of drug resistance [84].

## 4. Conclusions

Prdxs are no longer regarded as simple peroxide-scavenging enzymes. Rather, through reversible structural and functional transitions, they function as moonlighting proteins involved in antioxidant defense, peroxide sensing, signal transduction, and proteostasis [59,85].

An important concept is that Prdx function is regulated by PTMs. In addition to classical regulatory mechanisms such as phosphorylation and acetylation, diverse PTMs, including glutathionylation, S-nitrosylation, S-sulfhydration, sumoylation, ubiquitination, and lactylation, have expanded the regulatory scope of Prdxs [12]. These modifications regulate peroxidase activity, oligomerization, protein–protein interactions, and degradation pathways, enabling Prdxs to function as central hubs that coordinate redox signaling in a context-dependent manner [86,87,88].

Moreover, individual Prdx isoforms exhibit distinct sensitivities depending on their oxidation state and PTM profiles, resulting in divergent biological responses under similar redox conditions. This specificity indicates that Prdx-mediated redox regulation cannot be uniformly applied across all cell types or disease states and must be interpreted within defined spatial and regulatory contexts [89,90].

These findings indicate a paradigm shift away from approaches focused on antioxidant activity toward strategies that directly modulate Prdx function. Selective regulation of Prdx catalytic states, PTM profiles, or disulfide bond-mediated protein–protein interactions offers the potential for more precise control of redox signaling. Collectively, future studies aimed at defining isoform-specific PTMs and their spatiotemporal functions will play a critical role in advancing Prdxs as biomarkers and therapeutic targets.

## Figures and Tables

**Table 1:** Basic features of mammalian Prdx isoforms.

Isoform	Subfamily	C_P_	C_R_	Disulfide Bond	Subcellular Localization
Prdx1	Typical 2-Cys	52	173	Intermolecular	Cytosol, nucleus, plasma membrane
Prdx2		51	172		Cytosol, nucleus, plasma membrane
Prdx3		108	229		Mitochondria
Prdx4		87	208		ER, extracellular space
Prdx5	Atypical 2-Cys	47	151	Intramolecular	Cytosol, mitochondria, peroxisome
Prdx6	1-Cys	47	–	–	Cytosol, lysosome

## Data Availability

No new data were created or analyzed in this study. Data sharing is not applicable to this article.

## Abbreviations

The following abbreviations are used in this manuscript:

aiPLA_2_	Acidic Ca^2+^-independent phospholipase A_2_
ATP	Adenosine triphosphate
C_P_	Peroxidatic cysteine
C_R_	Resolving cysteine
CSE	Cystathionine γ-lyase
COX1	Cyclooxygenase-1
DAMP	Damage-associated molecular pattern
ER	Endoplasmic reticulum
ERK	Extracellular signal-regulated kinase
IL-1β	Interleukin-1 beta
IVF	In vitro fertilization
FCD	Focal cortical dysplasia
Grx1	Glutaredoxin 1
GSH	Glutathione
GSTπ	GSH S-transferase pi
GPx	GSH peroxidase
GSDMD	Gasdermin D
GSNO	S-nitrosoglutathione
H_2_O_2_	Hydrogen peroxide
HMW	High-molecular-weight
HDAC6	Histone deacetylase 6
HCC	Hepatocellular carcinoma
LPS	Lipopolysaccharide
MOF	Males absent on the first
NF-κB	Nuclear factor kappa-light-chain-enhancer of activated b cells
NLRP3	NOD-, LRR- and pyrin domain-containing protein 3
NO	Nitric oxide
NRF2	Nuclear factor erythroid 2–related factor 2
NASH	Non-alcoholic steatohepatitis
ONOO^−^	Peroxynitrite
OSCC	Oral squamous cell carcinoma
PTP	Protein tyrosine phosphatase
Prdxs	Peroxiredoxins
PTM	Post-translational modification
PTK	Protein tyrosine kinase
PTX	Paclitaxel
RA	Rheumatoid arthritis
RNS	Reactive nitrogen species
ROS	Reactive oxygen speciesRGF: Regorafenib
SNOC	S-nitrosocysteine
STAT	Signal transducer and activator of transcription
Srx	Sulfiredoxin
TNF-α	Tumor necrosis factor alpha
Trx	Thioredoxin
TrxR	Thioredoxin reductase
ZNF207	Zinc finger protein 207
